# Public Opinion Early Warning Agent Model: A Deep Learning Cascade Virality Prediction Model Based on Multi-Feature Fusion

**DOI:** 10.3389/fnbot.2021.674322

**Published:** 2021-05-28

**Authors:** Liqun Gao, Yujia Liu, Hongwu Zhuang, Haiyang Wang, Bin Zhou, Aiping Li

**Affiliations:** Software Engineering Center, College of Computer, National University of Defense Technology, ChangSha, China

**Keywords:** agent system, deep learning, cascade virality prediction, feature fusion, classification

## Abstract

With the rapid popularity of agent technology, a public opinion early warning agent has attracted wide attention. Furthermore, a deep learning model can make the agent more automatic and efficient. Therefore, for the agency of a public opinion early warning task, the deep learning model is very suitable for completing tasks such as popularity prediction or emergency outbreak. In this context, improving the ability to automatically analyze and predict the virality of information cascades is one of the tasks that deep learning model approaches address. However, most of the existing studies sought to address this task by analyzing cascade underlying network structure. Recent studies proposed cascade virality prediction for agnostic-networks (without network structure), but did not consider the fusion of more effective features. In this paper, we propose an innovative cascade virus prediction model named CasWarn. It can be quickly deployed in intelligent agents to effectively predict the virality of public opinion information for different industries. Inspired by the agnostic-network model, this model extracts the key features (independent of the underlying network structure) of an information cascade, including dissemination scale, emotional polarity ratio, and semantic evolution. We use two improved neural network frameworks to embed these features, and then apply the classification task to predict the cascade virality. We conduct comprehensive experiments on two large social network datasets. Furthermore, the experimental results prove that CasWarn can make timely and effective cascade virality predictions and verify that each feature model of CasWarn is beneficial to improve performance.

## 1. Introduction

Currently, the number of agents is increasing rapidly (ichocki et al., 2011), and smart agents are more efficient. With the advancement of artificial intelligence technology, more and more intelligent agents are being used in the industry. A deep learning model provides a potential solution for artificial intelligence, it is widely used in various agents fields (Westerlund, 2020). With the rapid development of the Internet, the growth rate of information in online social networks has become an evaluation indicator of public opinion. Some information in the network will become the source of viral dissemination, and this information will spread like a storm. Different industries need to monitor their own network public opinion, especially for government, enterprises, and media industries. They pay attention to the impact of sudden public opinion on themselves. In other words, they want to know what information related to their own will become a viral cascade as early as possible (Tatar et al., 2014). Information cascade virality means that some information may be widely spread in the network in a short time. It may be organized, planned behavior, or the extension of controversial social emergencies (Kefato et al., 2018).

However, the cost of manually studying and judging information cascade virality is enormous. Automatically distinguishing early warnings through agents becomes a way to reduce labor costs in different industries. Designing efficient neural network algorithms to meet agents' needs to predict information cascade virality becomes the focus of research. Supposing this agent can use the features of significant differences and accurately warn when information becomes viral in the early stage. In that case, it plays a crucial role in the decision-making (blocking or guiding the dissemination) of the follow-up information dissemination. With the continuous progress of machine learning models, many advanced models apply information cascade scale prediction. These works use the network structure features of information dissemination and network nodes attribute features to establish a strong correlation, for example, the number of followers/followers of participating users, user connections and community structure, and user activity, etc. They mainly use machine learning models to predict the magnitude of the information forwarded at the future moment (Li et al., 2017, 2018; Wang et al., 2018). The emergence of deep learning technology improves manual feature selection in early work and obtains more high-dimensional space representation capabilities. However, in the field of cascade virality prediction, there are still two types of problems in previous works.

Firstly, a social network is usually scattered. Most of the previous virality predictions are based on many underlying social relationships (Subbian et al., 2017). It causes most models to rely on the underlying network features and uses users' network relationships to predict the cascade virality. However, it may be difficult to obtain such detailed network information in most cases. Besides, for different industries that only pay attention to their own information, it is not significant to obtain global social network relation data, such as the following relationship between users. It is not well-supported in terms of data volume and algorithm efficiency.

Secondly, network relational data are dynamic and complex. When applying deep learning models to solve network relational data, it usually requires more complicated information aggregation work (like GNN Zhou et al., 2018) to embed network nodes' representation, which requires a large number of model parameters. Simultaneously, in an information cascade process, new nodes will also cause new node embedding problems after joining the network, which brings about the problem of continuous training of new parameter models (Qiu et al., 2018). However, for many practical purposes, the timeliness of the virality of the information is more critical. If we can predict the virality earlier rather than later, such predictions are useful.

Some studies expect similar results with less feature information. They ignore the underlying network structure features (Zhao et al., 2015). Subbian et al. (2017) propose an agnostic network-based method to reduce the network structure information in the information cascade process. Kefato et al. (2018) apply deep learning models to agnostic-network virus prediction, use the number of forwarding in the time sequence process as the feature, and use the CNN model to predict whether the information will explode. However, the construction model lacks critical features strongly related to the information cascade.

To solve these problems, we propose a cascade virality prediction model based on deep learning, named CasWarn. First, we segment an information cascade with time slices and extract the cascaded features in different time slices, including dissemination scale, emotional polarity ratio, semantic evolution features, and use advanced models to vectorize these features. Next, we design a module with two neural network modules to aggregate these features. The first module uses a convolutional neural network to aggregate the relations between different features and uses asynchronous patterns to learn the potential relations of different time-series features. The second uses a variant of a recurrent neural network to learn the semantic evolution relations in the cascade process. Then, we use the gradient descent algorithm to train the classification model. The main contributions of our work are as follows:

(1) We design an intelligent agent model to predict the cascade virality of social network information, which can be applied to public opinion monitoring for different industries. We only need to monitor the dissemination content and time of information related to different industries and do not need to care about the user relationship involved in the dissemination. Using a relatively small amount of information, it can quickly and effectively predict social network information virality.

(2) We propose an improved deep learning model, CasWarn, for cascade virality prediction based on time series. CasWarn extracts the key features of information dissemination from the agnostic-network and fuses these features through a deep learning model, which makes it more suitable for cascade virality prediction tasks.

(3) We conduct extensive experiments on two public datasets, and our results prove that CasWarn outperforms the latest benchmarks in many agnostic-network cascade virality prediction tasks. Simultaneously, compared with the state-of-the-art knowable-network model, we have achieved comparable performance under the premise of a less information parameter.

The rest of this paper is structured as follows. In section 2, we briefly review related works. Section 3 gives the formal definition of an information cascade event and information cascade sequence and defines the problem of cascade virality prediction, while section 4 details the proposed CasWarn model. In section 5, we discuss the experimental evaluation of CasWarn against previous state-of-the-art baselines. We conclude our work in section 6.

## 2. Related Work

With the continuous advancement of artificial intelligence technology, more and more deep learning models are deployed on agents to solve problems in different fields, such as visual recognition (Ruiz-del-Solar et al., 2018; Gu et al., 2021), behavior supervision (Quan et al., 2018; Ganesan et al., 2021; Jia et al., 2021), and artificial assistance (Xiao et al., 2020), etc. Deep learning is defined as a scientific field involving complex functions (for example, non-linear dynamics) to train a multi-layer neural network, embedding the data from the original, high-dimensional, multimodal state to the understandable state of the agent system (Goodfellow et al., 2016). Due to the flexibility and adaptability of neural networks, it is very suitable for agent systems (Ciresan et al., 2010), especially at the most active research frontier, to help researchers study agent systems' perception capabilities (Marsland, 2009).

In the work of social network public opinion supervision, intelligent auxiliary agents can help different industries perceive their own network evaluations, and prompt the industry to follow-up or block the information. Many models based on deep learning have emerged in this field, and most of the work is to predict the scale of the information cascade through the model (Tsur and Rappoport, 2012; Jenders et al., 2013; Cheng et al., 2014; Weng et al., 2014; Gao et al., 2015; Zhao et al., 2015; Li et al., 2017). They use large-scale cascade indicators for intelligent early warning, and the main focus is predicted performance indicators and timeliness indicators (early detection). In order to obtain the macro-level predictive value of information cascade in social networks in time, many works decided to use machine learning models and divide the scale of cascade into two types of tasks: One uses regression, like (Kefato et al., 2018; Zhu et al., 2018), to predict the potential scale of an information cascade, and the other uses the classification models, such as Zhao et al. (2015), to define the form of dissemination as viral or non-viral. Based on the machine learning models, most of the work focuses on the following information cascade features: (a) network topology (e.g., user relationship, first-order relationship network structure of user, etc.); (b) network node features (e.g., user features, discussion content, information sources, or key early dissemination participants, etc.); (c) temporal features (e.g., forwarding interval, etc.).

In an information cascade influencing factors, some studies suggest that user features play a crucial role in the information cascade. One of the most common features is the number of followers. As the representative of user influence, it means that key users affect the speed and timing of the future dissemination scale (Zaman et al., 2013). Those who have many followers, such as celebrities and news industries, are more likely to have a larger number of cascading effects than ordinary users because their information is more evident in the network (Suh et al., 2010; Bakshy et al., 2011; Jenders et al., 2013). However, a large-scale information cascade is not only generated by influential users, but also closely related to the content of the information, and it makes sense to study large-scale cascades generated by ordinary users rather than celebrities (Dow et al., 2013). Some studies confirm that the text semantic contained in the information cascade process is considered one of the internal driving forces and key factors leading to cascade virality (Dong et al., 2015, 2016). Moreover, semantic features have better performance (with higher content complexity) in the cascade of observation topics (such as hashtags). For example, breaking news, rumors/fake news, hotspots, controversies/special topics, etc., attract more attention than normal content (Yano and Smith, 2010; Yan et al., 2011). Simultaneously, a lot of work confirms that in the process of an information cascade, user emotions involved in dissemination are important factors affecting information dissemination (Stieglitz and Dang-Xuan, 2012; Chen et al., 2016; Yuan et al., 2016). Pfitzner et al. (2012) introduce the concept of emotional divergence, which combines the positive and negative points of emotion in a tweet, and can also predict the probability of a tweet post being forwarded. In general, tweets with high emotional diversity are more likely to be retweeted, which affects the spread of the information. Jenders et al. (2013) on the relationship between emotional divergence and retweet probability can also confirm the research results of Pfitzner et al. (2012), and the sentiment analysis task is significantly improved through deep learning models. Tian et al. (2020) introduce sentiment knowledge to enhance pre-training (SKEP), learning a unified emotional expression and achieving better performance. Some preliminary and reliable attempts to explore network agnostic methods proved that useful and timely predictions could be made only based on the information learned from the cascade itself without any other network structure information (Subbian et al., 2017).

As mentioned, recent models in this field apply a neural network framework like CNN (Kefato et al., 2018), RNN (Li et al., 2017), and GNN (Chen et al., 2019), etc. By using the above information cascade features, the neural network model's excellent feature extraction ability is efficient for agent systems. Moreover, the semantic information in vector space can be better obtained by embedding the semantic information in the representation, such as word2vec (Goldberg and Levy, 2014).

For the reasons above, we use the classification method of agnostic-network. The advantage is that it is relatively “cheap” to obtain information dissemination for different industries (no need for global relationship structure or to construct a complex relationship network). We use the temporal features of an information cascade and process relatively easy-to-obtain text semantics, emotions, and dissemination scale, which are widely regarded as key features. Furthermore, we construct a timing-based sequence based on these features and propose a neural network feature fusion method, which obtained performance results comparable to expensive operations (such as the knowable-network models).

## 3. Model Framework

### 3.1. Problem Formulation

The problem of predicting the scale of each cascaded forwarding depends on the definition of virality. The most common and useful definition is its size (Cheng et al., 2014). For most practical purposes, it is much more difficult to predict the exact size than to know whether the cascade will be larger than a certain threshold. The threshold can be set as a relative measure or an absolute measure. Relative measure is used when the cascade size is unknown and relative to the population observed in the latest data (if user engagement with the social network is changing). In our task, we need to detect new information according to the needs of different industries, so we use the method based on fixed thresholds to predict the cascade's virality according to the task's needs.

***Definition 3.1.1 (Information Cascade Event)*** In the cascade virality prediction process of agnostic-network, we can regard the information cascade process as a event. It is constantly changing with the evolution of time. When we divide the event according to the timestamp, an information cascading event can be expressed as: *E* = [*e*_*t*_0__, *e*_*t*_1__...*e*_*t*_*n*__], where *e*_*t*_*i*__ is the state of the cascade when the event occurs at time *t*_*i*_, which is:

(1) $$\begin{array}{l} e_{t_{i}}=\left\{f_{j}^{t_{i}}:f_{j}^{t_{i}}\in R^{n\times m},i,j,m,n\in N\right\} \end{array}$$

where $f_{j}^{t_{i}}$ is the feature representation of the event at event *t*_*i*_ and *j* represents different feature types, which can be represented as normalized visible features. In this paper, we use three key features that cause cascade virality, semantic features, and local and emotional ratios. Section 3.2.1 introduces these features in detail.

***Definition 3.1.2 (Information Cascade Sequence)*** When an event is split into a series of sub-events, we define *E*(*t*_*s*_, *t*_*e*_) = [*E* : *E* ∈ (*E*_*t*_*s*__...*E*_*t*_*e*__)]. For the sake of brevity, we simplify the writing of *E*(*t*_*e*_): *E*(*t*_*e*_) = *E*(*t*_0_, *t*_*e*_), which means that the sub-events all start from *t*_0_. Through the above definition, we can get a cascade sequence of information as follows:

(2) $$\begin{array}{l} C=\left\{E\left(t_{0}\right),E\left(t_{2}\right),....E\left(t_{n}\right)\right\}. \end{array}$$

After that, we define two cascade sequences: *C*_*O*_*t*__ and *C*_Δ_*t*__, we consider *C*_*O*_*t*__ to be an observable sequence of the event, that is *C*_*O*_*t*__ = *C*(*t*_*n*_), which represents the set of sub-events of an event from the start time *t*_0_ to *t*_*n*_, and *C*_Δ_*t*__ = *C*(*t*_*n*_ + Δ_*t*_) is considered to be an unobservable sequence.

***Problem 1: Cascade Virality Prediction*** In order to better solve the problem of cascade virality prediction, according to the above definition, we can obtain an observable event subsequence *C*_*O*_*t*__, which contains each event slice *E*_*i*_ and cascade features *e*_*t*_*i*__ within the time slice. We seek to predict the magnitude of the information cascade event; $C_{\Delta_{t}}^{Num}=|C_{\Delta_{t}}|$ is larger than the absolute threshold τ in the prediction time Δ_*t*_. Specifically, given a cascade *C*_*O*_*t*__ and a absolute threshold τ ∈ *N*, if $C_{\Delta }^{Num}\geq \tau$ then *C*_*O*_*t*__ is labeled as a viral cascade. That is, we need to quantify the activation probability of information virality after the time interval Δ_*t*_, which is denoted as follows:

(3) $$\begin{array}{l} P_{v}=P\left(C_{\Delta_{t}}^{Num}|C_{O_{t}}\right). \end{array}$$

Where *P*_*v*_ is the probability of whether the cascade is viral or not, and *C*_*O*_*t*__ is the cascade sequence containing features. Further, the cascade virality prediction can then be formulated as, given *C*_*O*_*t*__, Δ_*t*_, finding an optimal mapping function L that minimizes the following objective with parameters Θ:

(4) $$\begin{array}{l} L\left(\Theta \right)=-\overset{N}{\underset{i=1}{\sum }}\log P_{\Theta }\left(C_{\Delta_{t}}^{Num}|C_{O_{t}};\Theta \right). \end{array}$$

### 3.2. CasWarn Model

We hope to use the key features to predict the possibility of a viral cascade under the premise of the least available data and return the prediction results in the form of early warning to realize the early warning agent. Through the previous work of Subbian et al. (2017), we know that the viral cascade may begin to spread rapidly in the first few hours. In contrast, a non-viral cascade takes a long time to reach a small number of users.

Unlike the previous network-agnostic cascade virality prediction models, we consider that more features can be used, but the challenge is fusing and embedding features. In addition to statistical features such as the dissemination time, dissemination scale, etc., many useful features can be obtained, such as text and tweets' emotional features. We know that these features significantly impact the information cascade's virality and apply to agnostic networks from previous studies. Based on the above assumptions, we use deep learning models to model a time series-based multi-feature cascade prediction model. In summary, the model construction process is as follows:

1. For each information cascade process *C* in our dataset, we extract *C*_*t*_*n*__ in the observable time window, where *t*_*n*_ is the end of the timestamp we can observe.

2. We label the cascade *C* and determine whether it is viral or not by threshold τ at time to Δ_*t*_.

3. Regarding whether the cascade sequence *C* will become viral, we segment the cascade based on a time window, and use different embedding methods to extract the dissemination scale, semantic evolution feature, and emotional polarity ratio features in different time slices, then use them as the input of the neural network model, and predict the probability as a classification task.

#### 3.2.1. Data Preprocessing

We slice the observable cascade sequence *C* based on time series and sample different features with equal time windows. In the time slice, we extract the cascaded features through the following three steps:

***a) Dissemination scale feature***

**Figure 1:**
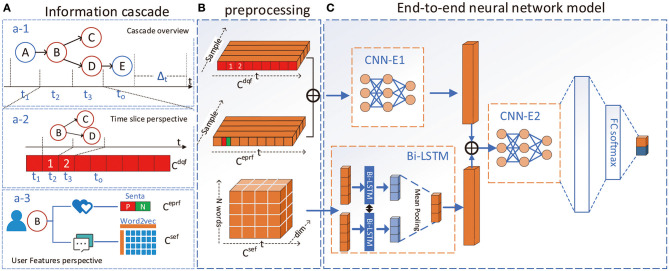
The overall architecture of CasWarn. **(A)**: Three perspectives of information cascade. (a-1): The overall information cascade after time slice; (a-2): the composition form of dissemination scale feature after time slice; (a-3): features in the user's view, including emotional polarity ratio and semantic evolution features. **(B)**: Different feature preprocessing and embedding representation processes. **(C)**: The end-to-end neural network model. It first fuses the quantitative and emotional features through the CNN-E1 layer and then embeds the temporal semantic evolution features through the Bi-LSTM model, it next uses the CNN-E2 model to fuse the three features again. Finally, the FC-softmax layer predicts the result.

**Figure 2:**
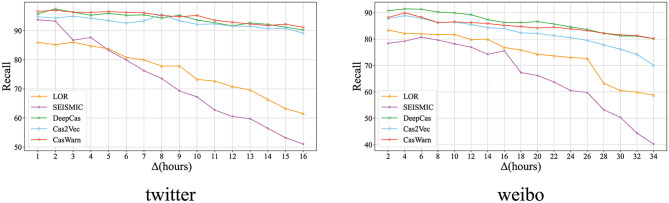
Evaluation results of early prediction experiments for the Twitter and Weibo datasets.

Dissemination scale feature is a crucial indicator to determine the virality of the cascade. We process it into a sequence by extracting the number of forwardings in the time slice, as shown in Figure 1a–2. Specifically, by extracting the forwarding times in the time slice, similar to Kefato et al. (2018), the sequence of integers representing the number of events included in each slice becomes:

(5) $$\begin{array}{l} C^{dpf}=\left[|C_{O_{t_{i}}}|:0\leq t_{i}<t_{o}\right] \end{array}$$

where *C*_*O*_*t*_*i*___ represents the total number of reposts at time *t*_*i*_, *t*_*o*_ represents the sequence observation time, and *i* represents the *i* − *th* time slice.

**Figure 3:**
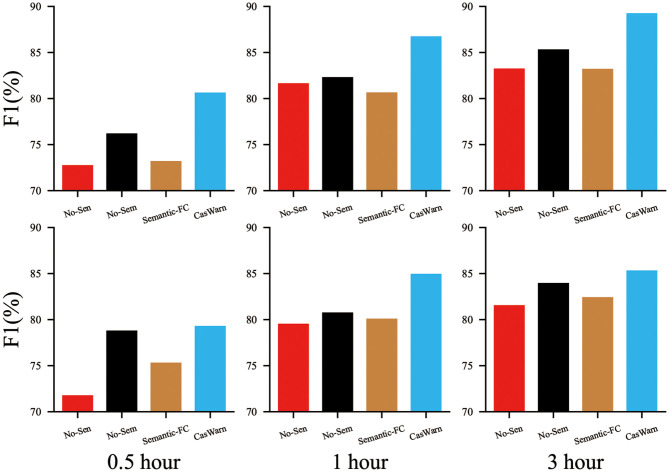
Performances of variant proposed models.

***b) Emotional polarity ratio feature*** We calculated the emotional polarity of users participating in the information cascade for forwarding and commenting in the time slice, as shown in Figure 1a–3. Specifically, we use the Senta model, which can obtain the comments' emotional polarity to the original tweets (Tian et al., 2020). Then we construct the emotional ratio in a time window. This ratio is a two-tuple, indicating the degree of opposition of emotions in the time window:

(6) $$\begin{array}{l} C^{eprf}=[\left(sum\left(p_{s}^{t_{i}}\right):sum\left(n_{s}^{t_{i}}\right),p_{s}=1,n_{s}=0,0\leq t_{i}<t_{o}\right] \end{array}$$

where *p*_*s*_ is positive emotion and *n*_*s*_ is negative emotion.

***c) Semantic evolution feature***

For viral cascades, the evolution of topic semantics is more likely to cause the “mutation.” By transforming topics in different time slices, we want to capture the impact of the features of topic evolution on the spread of the virus. We use the word2vec method to vectorize the semantic information of high-frequency topics in the time window, as shown in Figure 1a–3. Specifically, we extracted *topic*_*n*_ words with the highest word frequency in each time window to represent the key semantic features of this time window and form a matrix sequence based on the time series:

(7) $$\begin{array}{l} C^{sem}=\left[X^{t_{i}}:X\in R^{d\times n},0\leq t_{i}<t_{o}\right]\\ \;X^{t_{i}}=\left[T_{i}^{t}:0\leq i<topic_{n},T\in W\right] \end{array}$$

where *X* is the subject word in the time slice, *T* is the keyword, and *W* is the corpus. It is worth noting that if the number of samples in a specific time slice is less than *topic*_*n*_, we perform a zero-padding operation.

#### 3.2.2. Neural Network Model

After obtaining the three features, we design an end-to-end neural network model (Figure 1C) to predict the cascade virality. We use the CNN as the main framework to solve the prediction task. Because in the time series classification task, a CNN has the advantages of high efficiency and high performance (Gundersen et al., 2020), we changed the frame structure of the original CNN to better adapt to the task needs.

As shown in Figure 1C, we use two layers of CNN convolutional layers in the neural network model to obtain the feature representation after feature fusion. In particular, we have designed an improved two-way method for the representation of semantic features in the time window. The Bi-LSTM layer (Yulita et al., 2017) learns the potential connections in the topic evolution process and finally uses the fully connected layer to predict the results.

##### 3.2.2.1. Input Layer

As illustrated in Figure 1B, the input layer constructs a feature vector for three types of features. We can see from Figure 1C that these features are synchronized in the time series, but when input as a model, the steps are asynchronous.

##### 3.2.2.2. CNN-E1 Feature Fusion Layer

As shown in the CNN-E1 part of Figure 1C, we concatenate *C*^*dqf*^ and *C*^*eprf*^ as a vector into the convolution layer of the CNN model, as:

(8) $$\begin{array}{l} h_{c}=\left[h_{c^{dqf}}||h_{c^{eprf}}\right]. \end{array}$$

It is worth noting that we want to obtain the implied features of filters with different sliding windows, so we apply *n* filters of different sizes on every possible slice of the fused features:

(9) $$\begin{array}{l} f_{l}\left(h_{c}^{i}\right)=\sigma \left(W^{i}h_{c}^{i}+b^{i}\right) \end{array}$$

where $h_{c}^{i}$ can be regarded as a filter with step size i, *i* ∈ (1, *slice* − 1), σ is the activation function, and we use the relu method. Similar to the n-grams method, we can regard *W*^*i*^ as the weight of the filter layer of i^*th*^, and $h_{c}^{i}\in R^{kd}$ is the feature representation with the async length *i*. We sample the dependencies between subsequences by obtaining different convolutional layers. Then we obtain the fused feature representation by summation average:

(10) $$\begin{array}{l} f_{1}\left(h_{c}\right)=\frac{1}{i}\underset{i\in n}{\sum }f_{l}\left(h_{c}^{i}\right) \end{array}$$

where, $f_{1}\left(h_{c}\right)\in R^{d1}$, and *n* represents the number of filters.

##### 3.2.2.3. Bi-LSTM Semantic Evolution Layer

For the feature representation of semantic evolution, we design an improved architecture based on bi-directional LSTM (Bi-LSTM) to obtain the potential relationship of topic evolution in different time slices. Unlike the fully connected layer, it can capture the potential relations of semantic changes, as shown in the Bi-LSTM layer in Figure 1C. Firstly, since the number of keywords extracted in each time interval is fixed, we first concatenate the semantic information which represents the semantic feature vector in each time slice:

(11) $$\begin{array}{l} h_{c^{sef}}^{t}=|{|}^{i\in topic_{n}}h_{i}^{t} \end{array}$$

where $h_{i}^{t}$ represents the representation of the i-th feature word under the t-th time slice. Then, for different time slices, the content embedding of $h_{c^{sef}}$ is computed as follows:

(12) $$\begin{array}{l} f_{2}\left(h_{c^{sef}}^{t}\right)=\frac{\sum_{t\in slice}\left[\vec{LSTM}\left\{W\left(h_{c^{sef}}^{t}\right)\right\}||\overset{\leftarrow }{LSTM}\left\{W\left(h_{c^{sef}}^{t}\right)\right\}\right]}{\left|slice\right|} \end{array}$$

where $f_{2}\left(h_{c^{sef}}\right)\in R^{d\times 1}$ (d: content embedding dimension), *slice* represents the number of time slices, and W represents the learning parameters of neural networks. The operator || denotes concatenation. We use the Bi-LSTM model to learn the potential relationship of semantic evolution. The LSTM is formulated as:

(13) $$\begin{array}{r} {\text{z}}_{i}=\sigma \left(U_{z}\left(h_{c^{sef}}^{t}\right)+W_{z}{\text{h}}_{i-1}+{\text{b}}_{z}\right)\\ {\text{f}}_{i}=\sigma \left(U_{f}\left(h_{c^{sef}}^{t}\right)+W_{f}{\text{h}}_{i-1}+{\text{b}}_{f}\right)\\ {\text{o}}_{i}=\sigma \left(U_{o}\left(h_{c^{sef}}^{t}\right)+W_{o}{\text{h}}_{i-1}+{\text{b}}_{o}\right)\\ {\hat{\text{c}}}_{i}=\tanh\left(U_{c}\left(h_{c^{sef}}^{t}\right)+W_{c}{\text{h}}_{i-1}+{\text{b}}_{c}\right)\\ {\text{c}}_{i}={\text{f}}_{i}◦{\text{c}}_{i-1}+{\text{z}}_{i}◦{\hat{\text{c}}}_{i}\\ {\text{h}}_{i}=\tanh\left({\text{c}}_{i}\right)◦{\text{o}}_{i} \end{array}$$

where ${\text{h}}_{i}\in {\mathbb{R}}^{\left(d/2\right)\times 1}$ is the output hidden state of i-th content, ° denotes the Hadamard product, $U_{j}\in {\mathbb{R}}^{\left(d/2\right)\times d_{f}}$, $W_{j}\in {\mathbb{R}}^{\left(d/2\right)\times d_{f}}$, ${\text{b}}_{j}\in {\mathbb{R}}^{\left(d/2\right)\times 1}$, and (*j* ∈ {*z, f, o, c*}) are learnable parameters, *z*_*i*_, *f*_*i*_, and *o*_*i*_ are the forget gate vector, input gate vector, and output gate vector of the i-th semantic evolution feature, respectively. It is worth noting that the Bi-LSTM model can aggregate the ordered semantic information in order to obtain the implicit association of the semantic evolution process in different time slices.

##### 3.2.2.4. CNN-E2 Feature Fusion Layer

Next, we concatenate the semantic evolution feature $f_{2}\left(h_{c^{sef}}^{t}\right)$ with the output feature *f*_1_(*h*_*c*_) of the previous layer:

(14) $$\begin{array}{l} h_{des}=f_{1}\left(h_{c}\right)||f_{2}\left(h_{c^{sem}}\right). \end{array}$$

As shown in the CNN-E2 layer in Figure 1C, the concatenated data are fused again by the CNN feature fusion layer to learn the potential relationship between different features:

(15) $$\begin{array}{l} f_{3}\left(h_{des}\right)=\frac{1}{i}\underset{i\in n}{\sum }\sigma \left(W^{i}h_{des}+b^{i}\right). \end{array}$$

Then, *f*_3_(*h*_*des*_) is followed by a fully connected (FC-softmax layer) logistic classification layer:

(16) $$\begin{array}{l} h\left(c_{i}\right)=softmax\left(Wf_{3}\left(h_{des}\right)+b^{i}\right). \end{array}$$

The vector $h\left(c_{i}\right)\in {\mathbb{R}}^{2}$ can be regarded as the last feature representation in the model, which will be used to predict the virality of the cascade.

##### 3.2.2.5. Output Layer and Loss Function

This layer outputs a two-dimensional representation vector for each information cascade. We compare the representation of the dissemination scale feature with the ground truth, and then optimize the log-likelihood loss, as follows:

(17) $$\begin{array}{l} \min\underset{i}{\sum }y_{i}\log\left(h\left(c_{i}\right)\right)+\left(1-y_{i}\right)\log\left(1-h\left(c_{i}\right)\right) \end{array}$$

where *h*(*c*_*i*_) is the predicted value, *y*_*i*_ is ground truth, and the model parameters are trained using the back-propagation algorithm.

There are three main advantages for this framework: (1) It has concise structures with relatively low complexity (fewer parameters), making the model implementation and tuning relatively easy; (2) it can fuse the key features of information cascade in the agnostic-network and has a strong classification performance; (3) since the model is modular, it is flexible to add extra features, making the model extension more available.

## 4. Experiments and Results

In this section, we conduct extensive experiments to answer the following research questions: **(RQ1)** How does CasWarn perform the virus cascade prediction task compared with the state-of-the-art baselines? **(RQ2)** How does CasWarn compare with most state-of-the-art baselines in terms of early detection capability? **(RQ3)** How do different features, such as emotional polarity or semantic evolution, affect the performance of the model? **(RQ4)** How do various hyper-parameters, e.g., number of slices, word embedding dimension, impact the model performance?

### 4.1. Datasets

Our experiment selected two real-world social network datasets. One mostly exists to evaluate their methods of predicting diffusions on single social network data (Zhang et al., 2013, 2015; Cao et al., 2020). Another is a dataset of Twitter posts that we collected for specific semantics for different industries' needs to evaluate the proposed CasWarn model quantitatively.

#### 4.1.1. Weibo Dataset

Sina Weibo is the most popular Chinese Weibo service. The dataset is from (Zhang et al., 2013) and can be downloaded here. The complete dataset contained 1.78 million users and 23 million tweets between September 28, 2012 and October 29, 2012. It is worth noting that 300,000 original tweets in this dataset become information cascades. We sample these cascades, as shown in Table 1.

**Table 1:** Summary of dataset statistics.

**Dataset**	**Original tweets**	**Retweets average**	**Positive samples**	**Positive retweets**
Weibo	300,131	7.91	6,734	5,725,352
Twitter	470,435	4.43	9,723	7,786,556

#### 4.1.2. Twitter Dataset

During the unrest in Hong Kong in 2019, the government was more concerned about whether Hong Kong-related tweets would become viral cascades. Unlike Sina Weibo, different industries' social public opinion events are more aggregated at a semantic level. Therefore, in order to better verify the impact of fusion features on the information cascade, we collected anonymized tweets related to Hong Kong from September to October 2019 for a total of 30 days by using the Twitter API, to better verify the authenticity of the cascade virality prediction of the content we care about.

As Table 1 shows, we sample data with a cascade scale of more than 1,000 times (τ = 1, 000) as positive samples, negative samples are obtained by random sampling.

### 4.2. Comparison Methods

We compare CasWarn with a set of strong baselines, including feature-based models used for cascade prediction (Logistic Regression, SEISMIC), deep learning models based on cascade embedding in knowable networks (Deepcas), and the state-of-the-art deep learning models based on cascade embedding in agnostic networks (Cas2Vec).

**Logistic Regression (LOR)**: This baseline is used in previous studies. We concatenate the vector of each time window and calculate it as the LOR method's input for training a classification model. It should be noted that each time window contains the stitching of three feature vectors.

**SEISMIC**: This is a recent study that uses point estimation models to predict the popularity of tweets (Zhao et al., 2015). It evaluates the influence of tweets based on the number of retweets at time *t*, then the estimated infectiousness is used to predict the ultimate size of the tweet. We follow a similar strategy as Cas2Vec to label tweets based on fixed size, that is viral if and only if fixed size is larger than τ.

**DeepCas**: This is the state-of-the-art deep representation learning model for knowable-network popularity prediction (Li et al., 2017), which learns the representation of cascade graphs in an end-to-end model. Specifically, it represents the cascade graph. DeepCas significantly improves the performance of hand-crafting feature-based methods. As a result, we here take DeepCas as a knowable-network method to compare with CasWarn. Specifically, we use formula (17) to modify the loss function of DeepCas from regression task to classification task.

**Cas2Vec**: Cas2Vec is the state-of-the-art deep representation learning model for network-agnostic cascade virality prediction (Kefato et al., 2018). This model applies convolutional neural networks to model the sequence of retweet size within the time window and predicts information cascade virality.

### 4.3. Evaluation Settings and Implementation Details

To evaluate our algorithm, we use the following settings: As required by problem 1 (section 3.1), we want to predict the task based on the observable time *t*_*o*_ and the forecast time window Δ_*t*_. Since the distribution of viral cascades is highly skewed and sparse, we set the ratio of positive and negative samples to 1:2 and use validation sets during training to adjust hyper-parameters, such as the size of filters.

When the hyper-parameters are fixed, we use three-fold cross-validation without the validation set, and record the average results and errors. Regarding the embedding of topic words, we use a fixed value *topic*_*n*_ = 15 and use F-score with β = 3 (because it is a rare classification prediction).

### 4.4. Results

#### 4.4.1. Virus Cascade Prediction Performance (RQ1)

To answer RQ1, we design experiments to evaluate CasWarn on cascade virality prediction tasks. In this set of experiments, our goal is to evaluate the effective classification performance of our model under different baselines. We set the observable time *t*_*o*_ = 1 (1 h), and then evaluate F1 values under different Δ_*t*_ = *t*_*o*_ + Δ. The results are shown in the table.

Table 2 reports the performance of all models and shows the best results in bold. Comparing different baselines on the two datasets shows that the best baselines in most cases are our model. We can see that most of CasWarn's performance is better than the DeepCAS model based on the knowable-network. However, CasWarn is based on the agnostic-network, which requires less data and is more efficient. Compared with the agnostic-network model, the relative improvements of CasWarn over the Cas2Vec range from 1.3 to 2.3% and 1.5 to 6.0% for the Twitter and Weibo datasets. In general, the experimental results on the F1 value show that the deep learning framework we proposed is effective and demonstrates that it can outperform state-of-the-art baselines in the cascade virality prediction task of the agnostic-network.

**Table 2:** Prediction performance of different models on the two datasets (%).

**Model**	**Twitter**						**Weibo**					
	**1.5 h**	**2 h**	**5 h**	**10 h**	**20 h**	**60 h**	**1.5 h**	**2 h**	**5 h**	**10 h**	**20 h**	**60 h**
LOR	83.23	83.13	82.47	80.35	76.32	77.39	80.57	81.64	79.87	77.56	72.23	69.14
SEISMIC	82.61	79.77	77.26	74.53	72.24	59.57	77.44	75.32	72.21	69.57	62.27	60.50
DeepCas	86.97	87.42	**87.90**	83.21	82.77	**79.83**	83.74	**84.42**	83.21	**80.22**	79.38	76.77
Cas2Vec	85.98	86.72	85.43	84.35	82.78	77.21	83.26	83.33	**84.23**	74.57	73.31	72.29
CasWarn	**87.77**	**88.97**	87.45	**86.23**	**84.33**	78.77	**84.98**	82.21	83.44	79.90	**79.62**	**77.23**

*The bold values are represent the optimal performance values*.

#### 4.4.2. Early Prediction (RQ2)

In order to solve this problem, we analyze the prediction experiment of early information dissemination. And observed that most events occurred twice in the median time of spread of all cascade viruses. Similar to the work of Kefato et al. (2018), we selected the median of the viral cascade of two datasets. The median of Weibo is 16 h and Twitter is 7 h (due to Twitter data being more focused on topics, and that it is easier to quickly forward various pieces of network information). We choose a different (but fixed) prediction time Δ_*t*_ for each dataset, that is, 16 h for Twitter and 34 h for Weibo, and then change the size of the prediction window from 1 h to Δ_*t*_ h (the step is fixed, 1 h) to evaluate the time.

Note that the forecast time is fixed. In both cases, the step is 1 h. The rest of the hyper-parameters are the same as RQ1, but we use the recall rate to evaluate the performance of early detection.

Figure 2 shows that, as expected, our model achieves the best recall rate at the minimum value (1 h), which shows that we predict that the virus cascade's performance is the best within 1 h. At the same time, we hope that the prediction of the algorithm is robust as Δ_*t*_ increases. We can see that the performance of baselines declines faster than CasWarn, and CasWarn gets the best recall. In addition to the previous cascade virality prediction, our experiments also show that CasWarn is more robust than state-of-the-art models and can predict cascade virality as early as possible.

#### 4.4.3. Ablation Study (RQ3)

CasWarn is a framework for a deep learning early warning agent that fuses multiple cascade features. How different features impact the model performance and whether emotion polarity or semantic evolution aggregation effectively improves the model's predictive ability need to be addressed. To answer RQ3, we conducted an ablation study to evaluate the performance of several model variants, including: **(a)**: No-Sen which uses semantic evolution feature and dissemination scale feature encoding to represent each cascade sequence embedding (without emotional polarity feature). **(b)**: No-Sem which uses emotional polarity and dissemination scale feature encoding to represent each cascade sequence embedding (without semantic evolution feature), and **(c)**: Semantic-FC which employs a fully connected neural network to embed semantic evolution features.

In this group of experiments, we set the observation window as t0 = 0.5, 1, 3 (hours) and use the same parameter in RQ1 for our hyper-parameter. The results on two datasets are reported in Figure 3. From this figure, we can see that the performance of CasWarn is better than that of No-Sen in most cases, demonstrating that the cascade sequence embedded with the emotional polarity feature is more efficient for cascade virality prediction. Similarly, we can find that the performance of CasWarn is better than that of No-Sem, demonstrating that the fusion of the two cascade sequence features is effective in improving the performance of the model. Semantic-FC outperforms No-Sen, which shows that our improved Bi-LSTM-based semantic evolution feature embedding is better than the embedding methods used to capture “deep” content feature interactions such as the FC layer. It is worth noting that the two datasets show better results on the Twitter dataset due to the more aggregated semantic content of Twitter.

#### 4.4.4. Hyper-Parameter Sensitivity (RQ4)

**Figure 4:**
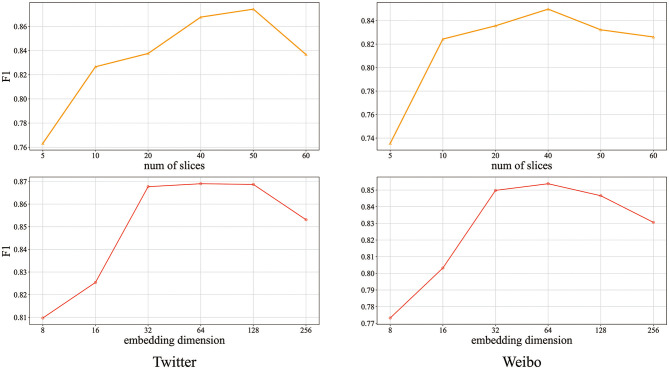
Impact of different hyper-parameters on prediction performance.

Hyper-parameters play an essential role in CasWarn because optimal parameters for cascade virality prediction determine the prediction results' accuracy. We conduct experiments to analyze the impacts of three key parameters, i.e., the number of slices (see section in the supplement for detailed setup) and the topic word's embedding dimension. Cascade virality predicts the performances of CasWarn as a function of the two datasets, which are shown in Figure 4.

For the number of slices parameter, from the upper part of Figure 4, we can see that performance improves as the number of slices increases and then drops at a peak. For the Twitter dataset, we can see that 40 to 50 is the best metric. In the Weibo dataset, the optimal solution is between 30 and 40. So we find that a value between 30 and 50 provides the best result. For the parameters of embedded dimensions of semantic evolution features, we set them to float before 16 to 256 dimensions. From the lower part of Figure 4, we can see that the performance is significantly improved with the increase of dimensions in the early stage. Then the performance has decreased with the increase of dimensions, which may be the result of over-fitting. The dimension when set to 32 meets the requirements of fewer variable parameters and timeliness.

## 5. Conclusions

In this paper, we propose a viral cascade early warning model, which can be deployed on intelligent agents to assist different industries in monitoring the public opinion effect of relevant information. Inspired by the agnostic-network cascade prediction, we design an innovative deep learning model based on feature fusion named CasWarn. This model serializes the cascade features through time slices, then fuses and embeds different key features through our designed neural network module, and then predicts the cascade sequence's virality. Our model incorporates the key features of the information cascade and does not need to consider the cascade network's underlying relationship structure, which is more suitable for the needs of fast, effective, and easy to deploy on agent systems. We conducted comprehensive experiments on two large social network datasets to prove that CasWarn can make timely and effective cascade virality predictions and verified that each feature model of CasWarn is beneficial to improve performance.

## Data Availability Statement

The original contributions presented in the study are included in the article/supplementary material, further inquiries can be directed to the corresponding author/s.

## Author Contributions

LG contributed to the core idea of the experiment design and analysis results under the guidance of BZ. YL assisted in the code for experiments and experiment analysis. HZ and HW analyzed the comparative experiment. BZ and AL supervised the research, provided financial support, provided the experimental equipment, and AL is the corresponding author. All authors discussed the results and contributed to the final manuscript.

## Conflict of Interest

The authors declare that the research was conducted in the absence of any commercial or financial relationships that could be construed as a potential conflict of interest.
